# Animal Model Dependent Response to Pentagalloyl Glucose in Murine Abdominal Aortic Injury

**DOI:** 10.3390/jcm10020219

**Published:** 2021-01-09

**Authors:** Jennifer L. Anderson, Elizabeth E. Niedert, Sourav S. Patnaik, Renxiang Tang, Riley L. Holloway, Vangelina Osteguin, Ender A. Finol, Craig J. Goergen

**Affiliations:** 1 Weldon School of Biomedical Engineering, Purdue University, West Lafayette, IN 47907, USA; ander934@purdue.edu (J.L.A.); eniedert@gmail.com (E.E.N.); retang@ucsd.edu (R.T.); hollowa2@purdue.edu (R.L.H.); 2 Department of Mechanical Engineering, University of Texas, San Antonio, TX 78249, USA; Sourav.Patnaik@UTDallas.edu (S.S.P.); vangelina.osteguin@my.utsa.edu (V.O.); ender.finol@utsa.edu (E.A.F.)

**Keywords:** abdominal aortic aneurysms, pentagalloyl glucose, ultrasound, elastin

## Abstract

Abdominal aortic aneurysms (AAAs) are a local dilation of the aorta and are associated with significant mortality due to rupture and treatment complications. There is a need for less invasive treatments to prevent aneurysm growth and rupture. In this study, we used two experimental murine models to evaluate the potential of pentagalloyl glucose (PGG), which is a polyphenolic tannin that binds to and crosslinks elastin and collagen, to preserve aortic compliance. Animals underwent surgical aortic injury and received 0.3% PGG or saline treatment on the adventitial surface of the infrarenal aorta. Seventeen mice underwent topical elastase injury, and 14 mice underwent topical calcium chloride injury. We collected high-frequency ultrasound images before surgery and at 3–4 timepoints after. There was no difference in the in vivo effective maximum diameter due to PGG treatment for either model. However, the CaCl_2_ model had significantly higher Green–Lagrange circumferential cyclic strain in PGG-treated animals (*p* < 0.05). While ex vivo pressure-inflation testing showed no difference between groups in either model, histology revealed reduced calcium deposits in the PGG treatment group with the CaCl_2_ model. These findings highlight the continued need for improved understanding of PGG’s effects on the extracellular matrix and suggest that PGG may reduce arterial calcium accumulation.

## 1. Introduction

Abdominal aortic aneurysms (AAAs) are a local dilation of the largest artery in the body. AAAs occur in the aorta below the diaphragm and typically below the renal arteries. AAAs are associated with significant mortality risk with about 10,000 deaths occurring in the U.S. annually [1]. Risk factors include age over 65 years old, male sex, and a history of smoking [2]. Since most AAAs are asymptomatic until rupture, annual screenings that assess the development and progression of AAAs are common. Once diagnosed, treatment is dictated by aneurysm size and growth rate. If dilation is less than 5.5 cm and growth rate is less than 1 cm/year, patients receive serial imaging to monitor the aneurysm. Aneurysms larger than 5.5 cm or with a growth rate greater than 1 cm/year require endovascular (EVAR) or open surgical repair as they are at a higher risk of rupture. Nonetheless, smaller aneurysms have been known to rupture, while many large aneurysms never rupture and may not require periodic monitoring [3].

AAA rupture is a serious event that requires urgent repair, which is associated with high mortality rates of 30–50% [4]. Elective repair is an option when patients are screened and know they have an aneurysm. These procedures carry lower 30-day mortality rates at 5% for open repair and 1–2% for EVAR but often require additional intervention and are not indicated for aneurysms smaller than 5.5 cm [5]. Currently, no proactive treatment exists for small aneurysms under surveillance, as current methods of elective repair cause more harm than good [6]. Thus, there is need for preemptive, non-invasive treatments to prevent aneurysm expansion and subsequent rupture.

Pentagalloyl glucose (PGG) is a polyphenolic compound that possesses the unique cross-linking ability to bind hydrophobic regions of collagen and elastin [7]. This cross-linking of extracellular matrix (ECM) proteins has led to a stabilized arterial microstructure of the abdominal aorta [8] and partial prevention of mechanical degradation in enzyme-treated arterial tissues [9,10]. Specifically, PGG has been utilized in rat [8,11,12,13], mice [14], and porcine [15,16] models of AAA to suppress the pathological expansion. Isenburg et al. [8,17] first reported on the periadventitial application of PGG in a rat CaCl_2_ model of AAA, which resulted in an overall reduction of arterial dilation. This led to PGG-loaded nanoparticle delivery in rat CaCl_2_-based aneurysms [11,12] and mouse elastase-based aneurysms [14]. In these studies, there was an observed reduction in macrophage infiltration [11,14], calcification [12], matrix metalloproteinases MMP-9 and MMP-2 expression [14], and AAA growth. Others have explored alternative delivery methods: Thirugnanasambandam et al. utilized the periadventitial application of PGG to CaCl_2_-induced rat aneurysms and found that it was able to lower biomechanical stresses and MMP expressions [13]. A more recent study by Schack et al. [18] used a rat elastase model to demonstrate preservation of the elastic lamina through intraluminal delivery of PGG via a drug-eluting catheter. 

In terms of large animal models, porcine animals have been utilized to understand the effect of PGG on the elastase aneurysmal model [15] and a combined elastase/collagenase/periadventitial CaCl_2_ model [16]. Both methods utilize a balloon catheter for expansion of the abdominal aorta and for the intraluminal delivery of PGG. Kloster et al. [15] found that PGG-treated pigs experienced a reduction in the external anterior–posterior diameter to typical physiological values, demonstrating this polyphenol’s ability to inhibit early AAA development. Simionescu et al. [16] suggest that intraluminal PGG delivery to medium-size aneurysms attenuated AAA diameter expansion. Although this combined chemical approach of porcine AAA model development for evaluating PGG shows promise, it is difficult to elucidate which biochemical pathways are suppressed or promoted by PGG application. Nevertheless, the majority of vascular PGG studies have focused on the rat periadventitial CaCl_2_ model, with limited exploration in other animal models of aortic injury. Consequently, we chose two mouse models of aortic injury—periadventitial CaCl_2_ injury and periadventitial elastase injury—as a result of the lack of investigation of the effects of PGG in this species and its many well-established aortic injury and aneurysm models [19]. In this study, we aimed to evaluate the hemodynamic and microstructural changes in aortic tissue due to the periadventitial administration of PGG on calcium-chloride-treated (CaCl_2_) and elastase-treated models of abdominal aortic injury.

## 2. Materials and Methods

### 2.1. Surgical Procedure

Eight to fourteen-week-old C57Bl6/J mice underwent laparotomy. A midline incision was made along the abdominal wall through the skin and linea alba. Digestive organs were moved to the side and the retroperitoneum was pulled apart to expose the infrarenal aorta. We gently separated the aorta from the inferior vena cava (IVC) and left the retroperitoneum on the IVC to ensure only the aorta was exposed to treatments. Initially, animals received a 15 min treatment of 0.3% PGG [8,10] or saline topically applied to the infrarenal aorta via gauze soaked in the solution. The PGG solution was made by dissolving 25 mg of PGG (Sigma Aldrich, St. Louis, MO, USA) in 8.33 mL of 0.9% NaCl. The injurious mechanism was subsequently applied. 

We utilized the topical elastase model adapted from Anidjar et al. and Bhamidipati et al. [20,21]. A solution of concentration 5.0 mg/mL was created by dissolving 5 mg of porcine pancreatic elastase (PPE, Sigma Aldrich) in 200 µL of 0.9% NaCl. Five µL of this solution was topically applied for five minutes to the infrarenal aorta of 17 mice (eight PGG-treated, nine saline-treated) to create small, stable aneurysms.

For the second model, we used the topical CaCl_2_ model based on the methods described by Gertz et al. [22]. Eight animals received PGG, while six received saline treatment as discussed previously. Then, 200 µL of 0.5 M CaCl_2_ (Sigma Aldrich) was applied to the adventitial surface of the infrarenal aorta with gauze for twenty minutes to induce aortic injury. 

**Figure 1:**
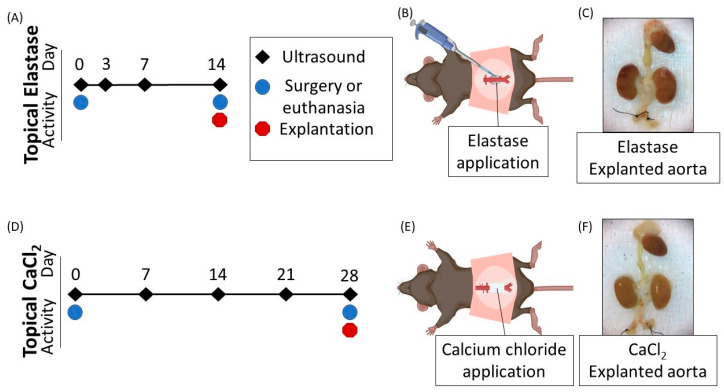
Timeline of experimental events. (**A**) Timeline for injuries induced by topical elastase. (**B**) Porcine pancreatic elastase was topically applied by pipette to the infrarenal aorta. (**C**) A representative aorta from the topical elastase group. Aortas were mechanically tested following explantation. (**D**) Timeline for injuries induced by calcium chloride (CaCl_2_). (**E**) CaCl_2_ was applied via gauze soaked in 200 µL of 0.5 M CaCl_2_. (**F**) A representative aorta from the topical CaCl_2_ group. Aortas were mechanically tested following explantation.

Following two weeks in the topical elastase group and four weeks in the CaCl_2_ group, we explanted the aortas and prepared them for mechanical testing (Figure 1A,D). Aortas were frozen at −80 °C for at least one week before being placed on dry ice for overnight transport for mechanical testing. All animals received free access to standard rodent food and water during the experiment. Qualified animal care staff cleaned and changed cages at least weekly. The Institutional Animal Care and Use Committee at Purdue University approved all procedures and experiments (protocol 1305000869).

### 2.2. Imaging Protocol

A high-frequency preclinical ultrasound system (Vevo3100, FUJIFILM VisualSonics Inc., Toronto, ON, Canada) was used to collect transverse and longitudinal images of the aorta. Animals were anesthetized using 1–2% isoflurane and placed on a heated imaging stage. Depilatory cream was applied to the abdomen to remove any animal hair. Ultrasound acoustic coupling gel was applied to the abdomen, and a linear array ultrasound probe (MX550D, FUJIFILM VisualSonics Inc.) with a center frequency of 40 MHz was used to follow aortic injury progression. We performed ultrasound imaging before surgery and every week thereafter. We followed animals for two weeks in the topical elastase model (where prior studies have shown no further expansion occurs after 14 days [23]) with an additional imaging session at three days post-injury. Animals were followed for four weeks in the CaCl_2_ model, as growth in this model can take longer [24]. All images were taken at three locations along the infrarenal aorta, where proximal indicated the level of the left renal vein, distal indicated the level of the aorta immediately prior to the bifurcation, and middle indicated the largest cross-section between proximal and distal. In the event that bowel gas obscured the infrarenal aorta during imaging, we employed gentle abdominal massage using a cotton-tipped swab to move the bowel and obtain a better image. Animal temperature, respiration rate, and heart rate were monitored throughout imaging.

ECG-gated kilohertz visualization (EKV) images, a type of acquisition where frames from several cardiac cycles are reconstructed to create one high-resolution cardiac cycle, were taken and used to calculate in vivo effective maximum diameter using Equation (1),

(1) $$Effective\;diameter=2\sqrt{\frac{\left(A_{S}+2A_{D}\right)}{3\pi }},$$

where $A_{S}$ is the area of the lumen during systole and $A_{D}$ is the area of the lumen during diastole. Motion mode (M-mode) images were taken and used to calculate systolic and diastolic diameters for estimation of the Green–Lagrange circumferential cyclic strain using Equation (2),

(2) $$E_{\theta \theta }=\frac{1}{2}\left({\left(\frac{D_{s}}{D_{d}}\right)}^{2}-1\right),$$

where $D_{s}$ is the lumen diameter during systole and $D_{d}$ is the lumen diameter during diastole. We additionally acquired three-dimensional datasets at each time point using a stepper motor. VevoLab (FUJIFILM VisualSonics) was used to measure all ultrasound-based diameters and areas. SimVascular (open source three-dimensional modeling software, version 2020.04.06, http://simvascular.github.io/) and Blender (open source graphics software, version 2.90.1, Blender Foundation, https://www.blender.org/) were used to segment and create three-dimensional renderings [25].

### 2.3. Biomechanical Testing

**Figure 2:**
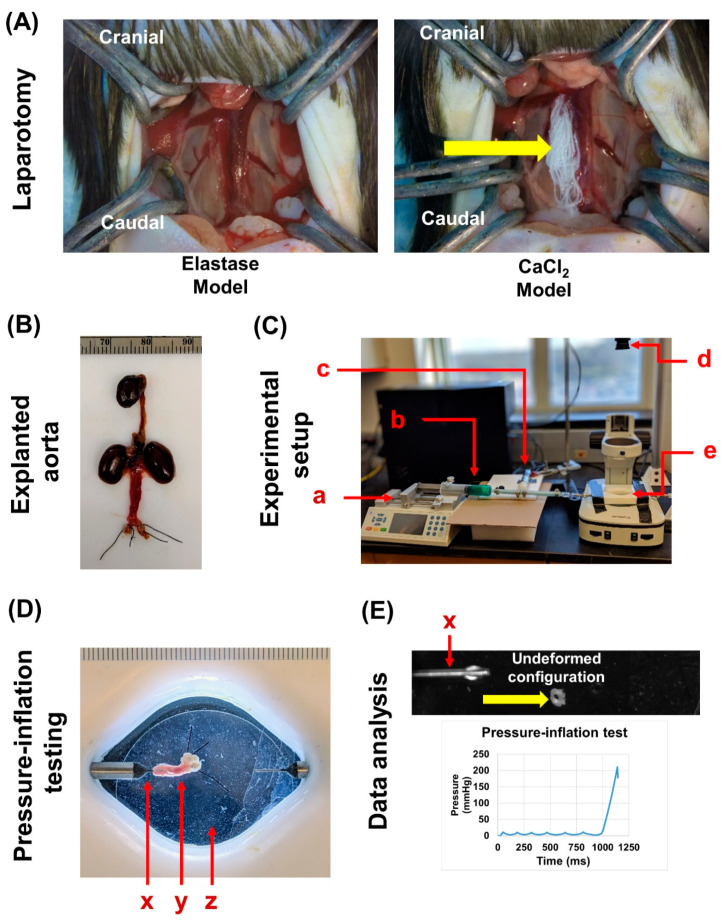
Biomechanical testing of abdominal aorta specimens. (**A**) Frontal views of the abdominal cavity of the elastase and CaCl_2_ models after laparotomy, respectively. The yellow arrow on the CaCl_2_ model indicates the CaCl_2_-soaked gauze that was applied to the periadventitial surface of the exposed abdominal aorta to initiate aortic injury. (**B**) Exemplary image of an explanted intact aorta along with the iliac arteries securely tied with sutures. (**C**) Experimental setup for pressure-inflation testing. The components are as follows: (a) Chemyx syringe pump, (b) syringe filled with PBS + food dye (for leak detection), (c) Omega pressure transducer with a range of 5 psi, (d) Basler high-resolution monochromatic camera for capturing specimen images, and (e) organ bath with 0.5 mm diameter cannula (World Precision Instruments). (**D**) Top view (as seen from the Basler camera) of the infrarenal aortic specimen (y), loaded onto the cannula (x), and placed in the saline filled organ bath (z). (**E**) To measure the thickness of the specimens, aortic rings were dissected (yellow arrow) and placed upright in the organ bath; the cannula (x) was utilized as a reference for image resolution, and the undeformed configuration (transverse view) was captured using the same camera setup. Using a custom LabVIEW script, incremental pressure changes with their accompanied time points were recorded for each specimen.

Abdominal aortas were excised from the aforementioned animals, and sutures were tied at the iliac bifurcation (Figure 2A,B). Extra fat or connective tissue was removed, and the specimens were stored at −80 °C until biomechanical testing was performed. Persons performing mechanical testing were blinded to specimen details. Complications in loading vessels for pressure-inflation testing resulted in some vessel tears, thereby preventing the mechanical testing of some specimens (for mechanical testing: elastase + saline *n* = 6, elastase + PGG *n* = 7, CaCl_2_ + saline *n* = 3, and CaCl_2_ + PGG *n* = 7).

We performed pressure-inflation testing using the “free-end” technique [26] to analyze the deformation of the vessels when subjected to incremental intraluminal pressures. Before testing, a small aortic ring segment (≈0.3 mm long) was dissected from the proximal end of the infrarenal aorta (Figure 2E). The cross-sectional view of this segment was photographed (Basler Inc., Ahrensburg, Germany) and quantification of the aortic wall thickness, and inner and outer radii were performed using Vision Assistant (National Instruments, Austin, TX, USA). These values represent the undeformed configuration of the aorta. Next, specimens were cannulated, i.e., the proximal end was mounted on the metal cannula (outer diameter of 0.5 mm; World Precision Instruments, Sarasota, FL, USA) and secured with a suture knot (Figure 2D) on a custom pressure-inflation testing device (Figure 2C). The distal ends, previously sutured, were suspended in room temperature phosphate buffered saline (PBS) solution (Millipore Sigma, Burlington, MA, USA) inside the device chamber at ≈ 25–27 °C (Figure 2D).

We performed intraluminal pressurization of the specimens using PBS solution mixed with food dye (for tracking leaks in the specimens) at a rate of 0.05 mL/min (syringe pump; Chemyx Inc., Stafford, TX, USA) (Figure 2C). Specimens were preconditioned with 6–8 pressure cycles ranging from 0 to 10 mmHg. Simultaneously, pressure data from a transducer (Omega Inc., Norwalk, CT, USA) (Figure 2C) and images were recorded every 0.5 s via a custom LabVIEW script (version 2019, National Instruments, Austin, TX, USA) throughout the duration of the test. We used a rate of 0.1 mL/min to pressurize the specimens until they burst, which was defined as the leakage of PBS through the vessel wall due to mechanical failure [27]. Pressure readings and image captures were synchronized and corrected up to 100 ms. We recorded burst pressure (mmHg) [28] and time-to-failure (s) [29] for each specimen (Figure 2E).

### 2.4. Histologic Preparation and Analysis

After mechanical testing, we processed ringlet specimens from each infrarenal aorta at the proximal, middle, and distal locations for routine histology. Specimens were submerged in 4% paraformaldehyde for at least 24 h, embedded in paraffin wax, sectioned on a microtome, mounted on a glass slide, and stained. Hematoxylin and eosin (H&E), Alizarin Red S (ARS), and Movat’s Pentachrome (MP) stains were applied to the arterial cross-sections for visualization of the general microstructure, calcium, and extracellular matrix proteins. Images were captured at 40× magnification using a VS120 Virtual Slide Microscope (Olympus Corporation, Waltham, MA, USA).

A certified pathologist blinded to specimen details performed a qualitative evaluation of the H&E slides.

#### 2.4.1. Microstructural Quantification

We further utilized the ARS and MP slides for semi-quantitative analysis of calcium and elastin contents, respectively. As in previous studies, [30,31,32], the elastin and calcium quantifications were performed as follows:

(3) $$\%\;Area\;=\;100\times \frac{chemical\;positive\;pixels}{chemical\;positive\;pixels+remaining\;constituents}.$$

We captured randomized regions of interest (ROIs) from multiple instances of the ARS and MP histology images for this purpose. In the MP images, elastin fibers were observed as blue-black, whereas other constituents were observed as lighter colors. The total tissue coverage for each ROI was quantified in pixels. Next, we converted the ROIs to gray-scale format (histogram from 0 = black to 255 = white) and applied Hue–Saturation–Balance (HSB) thresholding in NIH ImageJ [33] (specifically, brightness 0%—black through 100%—white) to isolate the elastin fibers from the rest of the constituents. Then, we quantified the elastin-positive pixels: using Equation (3), the area percentage of elastin was quantified by taking the ratio of elastin-positive pixels to total pixels multiplied by one hundred [34]. We repeated this step for multiple ROIs and recorded the mean value for each specimen. Similarly, the ARS-stained calcium nodules were observed as bright red, while the rest of the tissue section remained light pink to white. Using the same procedure described for elastin quantification, we quantified the amount of calcium in each ROI (derived from the ARS images), divided it by the total pixel area coverage per ROI, repeated the process for multiple ROIs, and reported the mean value for each specimen.

#### 2.4.2. Intima-Media and Adventitial Thickness

Utilizing the MP images, we measured the intima-media thickness (IMT) and adventitial thickness (AT) of each arterial cross-section, as reported in [35]. The shortest distance between the elastic fiber band and the luminal edge was considered the IMT. Conversely, the shortest distance between the periadventitial edge and the edge of the elastic fiber band was considered the AT. All measurements were performed on undamaged and artifact-free portions of the MP images for each specimen. Six to eight IMT and AT measurements were recorded per MP image and their mean value reported. Total thickness was calculated by the summation of IMT and AT for each specimen. All quantifications were performed using NIH ImageJ [33].

### 2.5. Statistical Analysis

Data from all four groups (elastase + saline, elastase + PGG, CaCl_2_ + saline, and CaCl_2_ + PGG) were expressed as mean values ± standard deviation for ultrasound, biomechanics, and histological parameters. For ultrasound data, we compared effective maximum diameter between groups and within models using a two-way repeated measures ANOVA with Šídák’s multiple comparisons (elastase + PGG vs. elastase + saline; CaCl_2_ + PGG vs. CaCl_2_ + saline). We likewise analyzed strain data in a similar fashion using a two-way repeated measures ANOVA with Šídák’s multiple comparisons, treating each location separately.

Biomechanical data, burst pressure, and time-to-failure for all four groups were compared between the PGG-treated and untreated counter parts using unpaired Student’s *t*-tests (elastase + PGG vs. elastase + saline; CaCl_2_ + PGG vs. CaCl_2_ + saline). Next, we performed a regression analysis between burst pressure and time-to-failure for all four groups, additionally comparing the obtained regression line characteristics (slope, intercept, etc.) with unpaired t tests.

Similarly, quantitative histological data (percentage elastase, percentage calcium, IMT thickness, AT thickness, and total thickness) were compared across the saline-treated and PGG-treated groups using unpaired *t*-tests.

We set the level of significance at *p* < 0.05 and performed all analyses using Prism (v.9.0.0, GraphPad Software, Inc., San Diego, CA, USA).

## 3. Results

### 3.1. In Vivo Imaging Revealed Preserved Green-Lagrange Circumferential Cyclic Strain in the PGG-Treated CaCl_2_ Group

**Figure 3:**
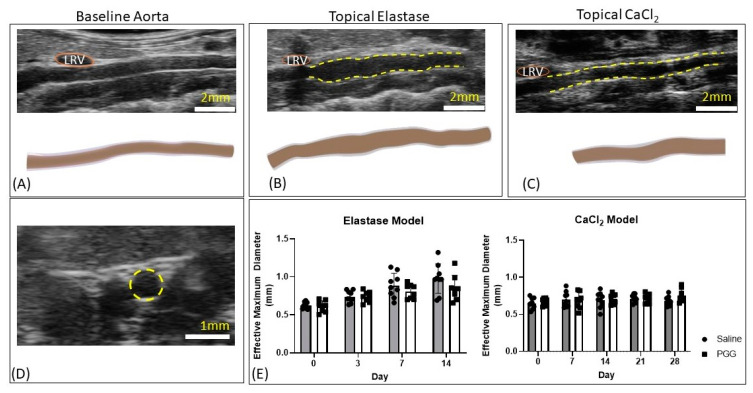
Diameter changes in the topical elastase and topical CaCl_2_ models. (**A**) Longitudinal ultrasound image and three-dimensional rendering of the baseline infrarenal aorta. LRV, left renal vein. (**B**) Representative longitudinal ultrasound image and three-dimensional rendering of an aorta from the topical elastase group 14 days after aortic injury. Yellow dashed lines indicate where treatment, either saline or PGG, was applied. (**C**) Representative longitudinal ultrasound image and three-dimensional rendering of an aorta from the topical CaCl_2_ group 28 days after aortic injury. Yellow dashed lines indicate where treatment, either saline or PGG, was applied. (**D**) Example short axis ultrasound image used to measure luminal area in systole and diastole to assess effective maximum diameter, as shown in Equation (1). (**E**) No differences between saline-treated and PGG-treated vessels were seen in either the elastase or CaCl_2_-treated vessels. Elastase-treated vessels increased in diameter over the two-week observation period.

No significant difference in effective maximum diameter was observed between PGG and the control treatment for either the topical elastase or topical CaCl_2_ models. The topical elastase model exhibited the expected expansion of the abdominal aorta, while the CaCl_2_ model did not show expansion (Figure 3). Qualitatively, the saline-treated CaCl_2_ vessels showed hyperechoic regions with reverberation artifact (an artifact common for calcium-based deposits and other strong reflectors) adjacent to vessel walls, while the PGG-treated group did not (Supplementary Video S1).

**Figure 4:**
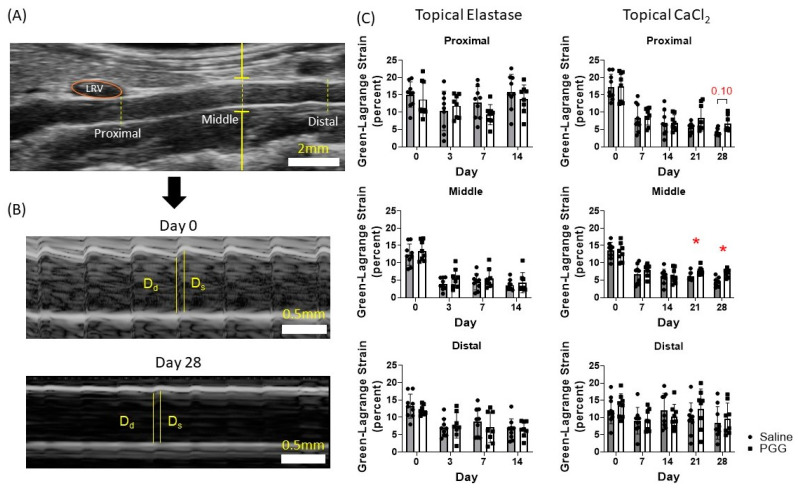
Green–Lagrange circumferential cyclic strain analysis. (**A**) M-mode acquisitions were taken at the proximal, middle, and distal infrarenal aorta. Representative locations are shown. LRV, left renal vein. (**B**) M-mode acquisitions at day 0 and at day 28 in the CaCl_2_-injured model demonstrating reduced compliance and measurement of the diastolic and systolic luminal diameters used to calculate Green–Lagrange circumferential cyclic strain, Equation (2). Systolic and diastolic measurements were made in triplicate and averaged, and the averages used to calculate Green–Lagrange circumferential cyclic strain. $D_{d}$, diastolic diameter. $D_{s}$, systolic diameter. (**C**) Green–Lagrange circumferential cyclic strain at each location in the topical elastase and CaCl_2_ groups. No difference in strain between saline or PGG treatment was seen in the topical elastase group. A statistically significant difference in strain was seen between treatments at the middle location in the CaCl_2_ group. Strain between treatments approached significance at the proximal location in the topical CaCl_2_ group.

The Green–Lagrange circumferential cyclic strain calculated from M-mode images demonstrated no significant difference between the PGG and control groups in the topical elastase model (Figure 4C). Interestingly, this was not the case in the CaCl_2_ model, where a statistically significant difference was seen at the middle location between the groups at day 21 (saline-treated 6.07 ± 1.16% vs. PGG-treated 7.72 ± 1.10%, *p* = 0.043) and day 28 (saline-treated 4.41 ± 1.28% vs. PGG-treated 7.11 ± 1.13%, *p* = 0.0017) and approaching significance at the proximal location at day 28 (saline-treated 4.24 ± 1.01% vs. 6.64 ± 2.28%, *p* = 0.10; Figure 4B). In other words, while both saline-treated and PGG-treated CaCl_2_ groups showed a decrease in Green–Lagrange strain from baseline, strain in the PGG-treated group was significantly higher than the saline-treated group at the end timepoint. No statistically significant difference was seen in either model at the distal location. Qualitatively, the CaCl_2_-induced injuries had lower strain at the proximal location compared to their elastase-induced counterparts.

### 3.2. Biomechanical Testing Demonstrated No Significant Difference in Burst Pressure or Time to Failure

**Figure 5:**
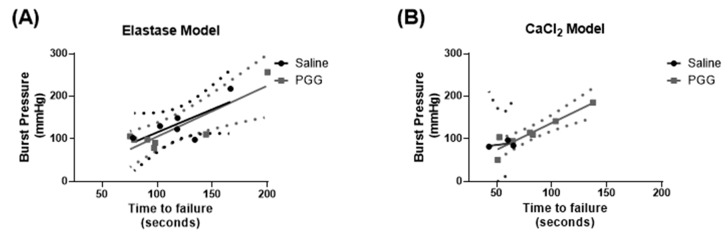
Ex vivo mechanical characterization of murine abdominal aortas. Biomechanical effect of PGG on elastase (**A**) and CaCl_2_ (**B**) injury models. No significant differences (*p* > 0.05 for both cases) were observed between the burst pressure (mmHg) vs. time-to-failure (s) profiles of control and PGG-treated specimens for both elastase and CaCl_2_ cohorts, respectively.

Pressure-inflation testing revealed no significant differences for specimens treated with saline vs. treated with PGG, respectively, in terms of burst pressure (elastase: 136.7 ± 43.9 mmHg vs. 120.0 ± 61.3 mmHg; *p* = 0.59) (CaCl_2_: 94.9 ± 9.4 mmHg vs. 118.9 ± 34.6 mmHg; *p* = 0.28) or time-to-failure (elastase: 119.6 ± 29.8 s vs. 112.2 ± 44.8 s; *p* = 0.74) (CaCl_2_: 58.7 ± 7.2 s vs. 80.6 ± 32.2 s; *p* = 0.29) across the two injury models. Furthermore, the overall slopes of the regression line between the two aforementioned, experimentally determined parameters resulted in similar slopes and intercepts (detailed in Table S1) across both the elastase and CaCl_2_ models (Figure 5A,B).

### 3.3. Histology Showed a Significant Difference in Calcium Deposition in the PGG-Treated CaCl_2_ Model

**Figure 6:**
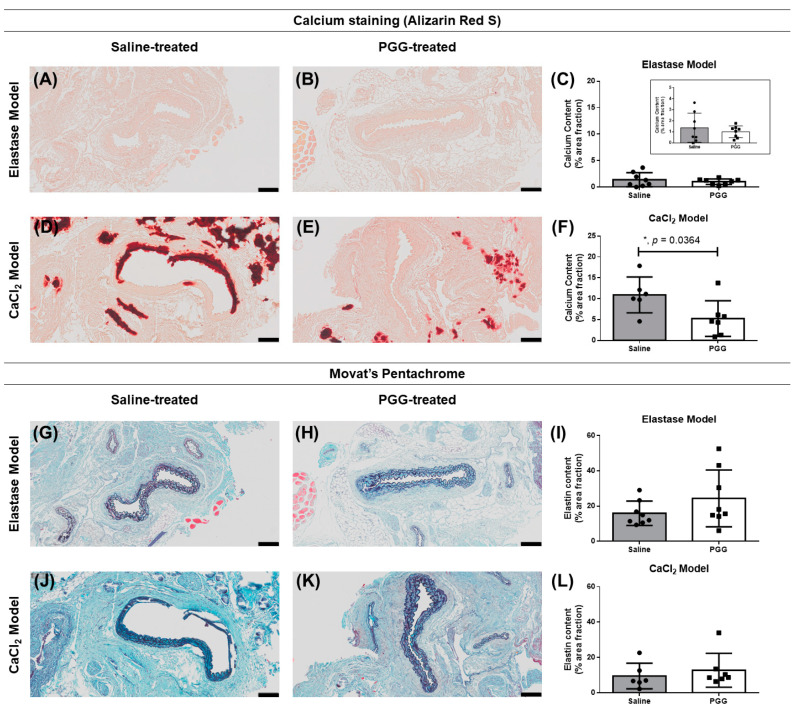
Histological characterization of abdominal aorta specimens obtained from elastase and CaCl_2_-treated animal models of aortic injury. Alizarin Red S-based staining and semi-quantitative estimation of calcium (dark red) in specimens from the elastase (**A**–**C**; inset is the zoomed in view of Figure 6C) and CaCl_2_ model (**D**–**F**). Movat’s Pentachrome stained sections and their respective semi-quantifications for elastin (dark violet to black) from all four groups, elastase + saline vs. elastase + PGG (**G**–**I**) and CaCl_2_ + saline vs. CaCl_2_ + PGG (**J**–**L**), are shown here. Calcium content was found to be significantly lower (almost twice) in the PGG-treated cohort as compared to their saline counterpart in the CaCl_2_-injured group (*p* = 0.0364), with almost no change in the elastase groups (*p* > 0.05; (**C**,**F**)). Elastin content was not significantly different between the saline-treated vs. PGG-treated cohorts of either injury models (*p* > 0.05; (**I**,**L**)). Scale bar = 100 µm.

Calcium content was not significantly different between saline-treated and PGG-treated animals in the elastase cohort (Figure 6A–C) (1.39 ± 0.47% vs. 1.02 ± 0.19%; *p* = 0.474). On the other hand, treatment with PGG led to a significant decrease (almost twice) in calcium content in the PGG-treated aortas compared to the saline-treated aortas in the CaCl_2_-injured group (Figure 6D–F) (10.91 ± 1.75% vs. 5.24 ± 1.61%; *p* = 0.0364). Furthermore, there was no difference in the semi-quantitative estimation of elastin between PGG-treated and saline-treated specimens for either the elastase (15.93 ± 2.45% vs. 24.37 ± 5.72%; *p* = 0.20) or CaCl_2_ group (9.54 ± 2.96% vs. 12.78 ± 3.63%; *p* = 0.51).

No significant difference was seen in IMT between PGG-treated and saline-treated aortas in either the elastase (28.59 ± 2.26 µm vs. 27.38 ± 1.44 µm; *p* = 0.424) or the CaCl_2_ model (28.94 ± 4.49 µm vs. 28.86 ± 2.07 µm; *p* = 0.99). Likewise, there was no significant difference in AT (elastase: 32.00 ± 5.96 µm vs. 30.92 ± 2.04 µm; *p* = 0.87) (CaCl_2_: 33.95 ± 3.11 µm vs. 35.57 ± 3.82 µm; *p* = 0.75) or total thickness (elastase: 61.58 ± 7.80 µm vs. 58.29 ± 3.02 µm; *p* = 0.70) (CaCl_2_: 62.89 ± 6.95 µm vs. 64.43 ± 4.89 µm; *p* = 0.86) for either group.

We found that the H&E images (Supplementary Section Figure S1) showed elastin fiber fragmentation that was prominently present in both the elastase and CaCl_2_ models. Some of the elastic fibers exhibited a straightening feature owing to the injury induced due to the initial insult with elastase or CaCl_2_, which is in contrast to their characteristic “wavy” crimped architecture. The elastase + saline specimens also exhibited some degree of necrosis in the tissue sections. On the other hand, the CaCl_2_ + saline specimens showed signs of chronic inflammation, macrophages, and fibrosis.

## 4. Discussion

The findings from this study highlight the complex nature of aortic tissue, the role of pathological ECM remodeling in AAA disease, and the need for additional work to explore biomechanical stabilization strategies of aneurysms. Higher strain in PGG-treated CaCl_2_ animals suggests that treatment affected vessel compliance. A difference in strain due to PGG treatment was not seen in the elastase model, suggesting that the mechanism of injury may play a role in the efficacy of PGG as a treatment for preventing pathological ECM remodeling. While no difference was seen in mechanical testing, histology revealed higher calcium content in saline-treated CaCl_2_-injured aortas compared to their PGG-treated counterparts. This suggests that an increase in calcium accumulation may contribute to a decrease in vessel compliance and thus the strain reduction we observed in vivo. As no animal model can recapitulate every aspect of the human disease, two common murine models of aortic injury were chosen here: the CaCl_2_ model relies on matrix metalloproteinases (MMPs) and inflammation to induce injury [24], while the elastase model takes a more direct, enzymatic approach, degrading elastin fibers in the intimal layer, while still inducing inflammation [21]. Stabilization of an in vivo strain using PGG in the CaCl_2_ model, but not the topical elastase model, suggests that ECM composition and quality may impact PGG efficacy, reinforcing the selection of an appropriate animal model as a key consideration for future studies.

From a biomechanics perspective, the medial layer of the arterial tissue bears the bulk of the pressure exerted by the blood flow, while the adventitial layer of the abdominal aortic tissue acts as a shield-like structure that prevents overstretching or rupture when the vessel is subjected to higher levels of pressure [36]. Burst pressure testing is a routine and robust evaluation for tubular biomaterials [37], and this approach is cohesive with the pathological pressure-laden rupture of AAAs. Burst pressure can be defined as the instance where a blood vessel fails as a result of pressure overload. Thus, any potential damage either to the elastin-rich medial layer (e.g., elastase-based injury) or to the helically oriented, collagen-based adventitial layer (e.g., CaCl_2_-based injury) would result in (i) weakening of the arterial microstructure and (ii) lower burst pressures during mechanical evaluations [38,39]. These weakening factors can be mitigated by vessel remodeling with increased collagen production and vessel thickening [40].

In this work, we investigated the ability of PGG to strengthen arterial tissue’s biomechanical properties due to this polyphenolic treatment. Previously, we demonstrated the ability of PGG to lower biomechanical stresses, via biaxial testing, and reduce the MMP expression levels in rat CaCl_2_ models of aortic injury (comparable to the mouse CaCl_2_ model in this study) [13]. In addition, we have also demonstrated biomechanical recovery, via biaxial tensile testing, of PGG-treated porcine aortic tissue specimens that were previously degraded with elastase and collagenase in vitro [9]. Following this, Pavey et al. reported in their in vitro study that prior PGG treatment of mouse carotid arteries prevented microstructural and mechanical damage by elastase, which was demonstrated by pressure-inflation testing [10]; however, they did not quantify the burst pressure of the specimens.

To evaluate the effect of PGG treatment on the burst pressure characteristics of the explanted aortas, we utilized the “free-end” mechanical technique per previously established studies, although with a modified pressure-inflation testing approach [26]. Burst pressure evaluation has been previously reported for 0.3% PGG-treated porcine carotid artery scaffolds [39] and 0.1% PGG-treated interstitial renal artery based tubular vascular grafts [41]. Although these studies are not directly comparable, the burst pressures reported in both did not show any significant differences between the PGG-treated constructs and their respective controls. This observation is similar to our findings, and we did not observe any significant effect of PGG’s cross-linking ability on the ex vivo mechanical characterization of arterial specimens obtained from either of the two experimental murine models. It is possible that the concentration of PGG utilized in our study (0.3%) is able to elicit a microstructural change in the animal models (i.e., lowering of calcium content in CaCl_2_ model; Figure 6) but not abundantly enough to inflict a biomechanical response. Furthermore, the periadventitial application of PGG relies heavily on the diffusion behavior of this compound. The diffusion characteristics of PGG via the adventitial side of the abdominal aorta is not fully understood, and it is likely a limiting factor in the functional crosslinking ability of this polyphenolic compound. To overcome this issue, a more controlled PGG-delivery approach, such as an external drug-eluting patch [7], could greatly improve the integration of the PGG to the arterial microarchitecture and potentially improve the mechanical properties of the injured arterial tissues.

In this regard, the method of PGG administration also appears to play a key role in treatment efficacy. The local administration of PGG to the adventitial surface of elastase- and CaCl_2_-treated murine abdominal aortas was performed in this present work, as previously reported animal studies showed a beneficial therapeutic effect [8,13]. However, direct periadventitial application would be technically difficult for patients not undergoing an open laparotomy and nearly impossible for patients treated with EVAR. Nonetheless, others have demonstrated PGG’s efficacy using nanoparticle-based approaches in rats [11,12] and surgical intraluminal pressure infusion in pigs and rats [15,16,18]. It is difficult to compare the different approaches to PGG delivery, as each method has its own merits and shortcomings. Nevertheless, the periadventitial approach is straightforward, avoids flow-based rapid washout, and produces no systemic toxicity [42]. Unfortunately, it also has limitations, including (i) lack of penetration to the elastin fibers in the media, (ii) no control of release kinetics, (iii) lack of uniformity around the circumference and length of the vessel, and (iv) local toxicity [43]. However, the initial aortic injury due to elastase and CaCl_2_ in our study originated from the outer surface of the abdominal aorta, and hence, PGG application on the same adventitial surface to illicit a counteracting effect seemed reasonable. Thus, both the improved delivery efficiency and an increased number of elastin lamellae may have provided greater potential for PGG binding and a reduction in ECM remodeling. Future work focused on clinical translation may need to consider the amount of elastin remaining in small AAAs and the most appropriate treatment strategy that minimizes risk while maximizing the delivery of stabilizing compounds.

Species size may also play a role in treatment efficacy in a laboratory setting. Here, we used mice due to the many well-established aortic injury and aneurysm models in this species. Still, it appears that PGG efficacy may depend on body size and initial aortic thickness, as studies demonstrating its efficacy appear to be more common in pigs and rats [8,11,12,13,15,16,18]. As the number of elastin lamellae in the aortic media increases allometrically with animal size [44], the typical mouse has only three to five layers in the abdominal aorta [45]. While our periadventitial elastase mouse model showed no differences between control and PGG-treated animals, others have shown PGG’s efficacy in a pressurized intraluminal infusion elastase rat model [18]. Overall, the findings presented here and in previous reports suggest that the experimental animal model, species size, and number of elastin lamellae should be considered carefully when evaluating biomechanical stabilization.

Beyond the animal model, quantity of elastin, and treatment approach, this study assessed the ability of PGG to prevent injury and did not quantify its efficacy on the abdominal aorta after damage. This is significant, as treatment for AAAs in the clinic is never performed on a healthy vessel. Rather, physicians carefully weigh the risks of monitoring versus intervention for each patient only once an AAA has been diagnosed and elastin is damaged. As mentioned above, elastin quantity and animal size may impact the efficacy of PGG, but the quality of remaining elastin may be just as important. While various delivery methods have been assessed, the majority use the CaCl_2_ model, which provides a slower-acting injury to elastin and collagen fibers [11,12,15,16]. We additionally used the topical elastase model to demonstrate the importance of model-specific differences. In this model, a faster-acting enzymatic injury proved to be too great a change for the PGG to counteract, emphasizing that elastin quality likely plays a role in PGG efficacy and highlighting the need for an improved understanding of the underlying mechanisms of PGG.

It is also worth noting that not all vessels in this study underwent sufficient expansion, which is defined as greater than 50% growth, to be considered an AAA. This may be a result of a difference in measurement methods—most prior studies report adventitial diameter upon dissection, using digital calipers or image analysis software [20,21,22,32,46,47,48,49]. In contrast, our study reports ultrasound-acquired luminal diameter at regular intervals up to and including the day of euthanasia. In the elastase model, while all vessels demonstrated growth, five of 17 vessels did not demonstrate enough growth to be considered aneurysmal. Nonetheless, vessel stiffening was observed in all mice. In the CaCl_2_ model, vessels did not demonstrably show luminal growth, but similar to the elastase model, a decrease in strain was seen in all vessels, which was tempered by PGG in the treatment group.

## 5. Conclusions

This study assessed the ability of PGG to preserve vessel biomechanics. While no change was seen between the treatment and control group in the elastase model, PGG-treated aortas in the CaCl_2_ model demonstrated preserved compliance through higher Green–Lagrange circumferential cyclic strain compared to their control-treated counterparts. Histology similarly revealed reduced calcium deposits in the PGG-treated CaCl_2_ group. These results highlight the heterogeneity of aortic tissue, the complex ECM remodeling process, and the need to further understand the roles that the animal model, quantity, and quality of elastin play when evaluating novel treatment strategies aimed at biomechanically stabilizing AAAs.

## Data Availability

The data presented in this study are openly available in the Purdue University Research Repository at doi:10.4231/1FQ0-P419.

## Supplementary Materials

The following are available online at https://www.mdpi.com/2077-0383/10/2/219/s1, Video S1: Ultrasound video of aortic compliance, Figure S1: Histological characterization of explanted abdominal aortas, Table S1: Statistical analysis of biomechanical data obtained from pressure-inflation testing.

## Institutional Animal Care and Use Committee Statement

All animal procedures were approved by the Purdue University Animal Care and Use Committee (Protocol 1305000869).
