# Supplementary material for: Animal Model Dependent Response to Pentagalloyl Glucose in Murine Abdominal Aortic Injury
Source: J Clin Med. 2021 Jan 9;10(2):219. doi: 10.3390/jcm10020219 (PMC7827576; doi:10.3390/jcm10020219)
Supplement: Supplementary file 1 [file jcm-10-00219-s001.zip › Supplementary section_Proofed.pdf]

Journal of Clinical Medicine

MDPI

Supplementary section

Video S1: Ultrasound video of aortic compliance

ECG-gated kilohertz visualization videos of an average cardiac cycle at baseline and for each group at end timepoints. Note the normal pulsatility observed in the baseline video. Similar pulsatility can be seen in the left-hand (cranial) side of each video as the injurious mechanism was not applied to this proximal region. Conversely, decreased pulsatility is noted just below the left renal vein in the middle section of the videos. This is consistent with the reduced strain values from baseline seen in Figure 4C. Elastase-injured vessels showed no difference in strain, but $CaCl_2$-injured vessels showed a significant difference in strain, with somewhat preserved compliance seen in PGG-treated vessels compared to saline-treated vessels. This is visible in the video: while the proximal infrarenal aorta (left hand side of each video) pulses normally, in the $CaCl_2$ group the middle section of the saline-treated vessel does not show expansion, rather it moves left to right. In contrast, the middle section of the PGG-treated $CaCl_2$-injured vessel expands, albeit it to a lesser extent than the representative baseline vessel. Qualitatively, hyperechoic reflectors with reverberation artifact (red arrowheads, this type of artifact is commonly seen with strong reflectors such as calcium deposits) were seen in the saline-treated $CaCl_2$ group. This corresponds well with histologic analysis where this group exhibited increased percent area calcium staining compared to the PGG-treated $CaCl_2$ group. Scale bar = 2mm.

Figure S1: Histological characterization of explanted abdominal aortas

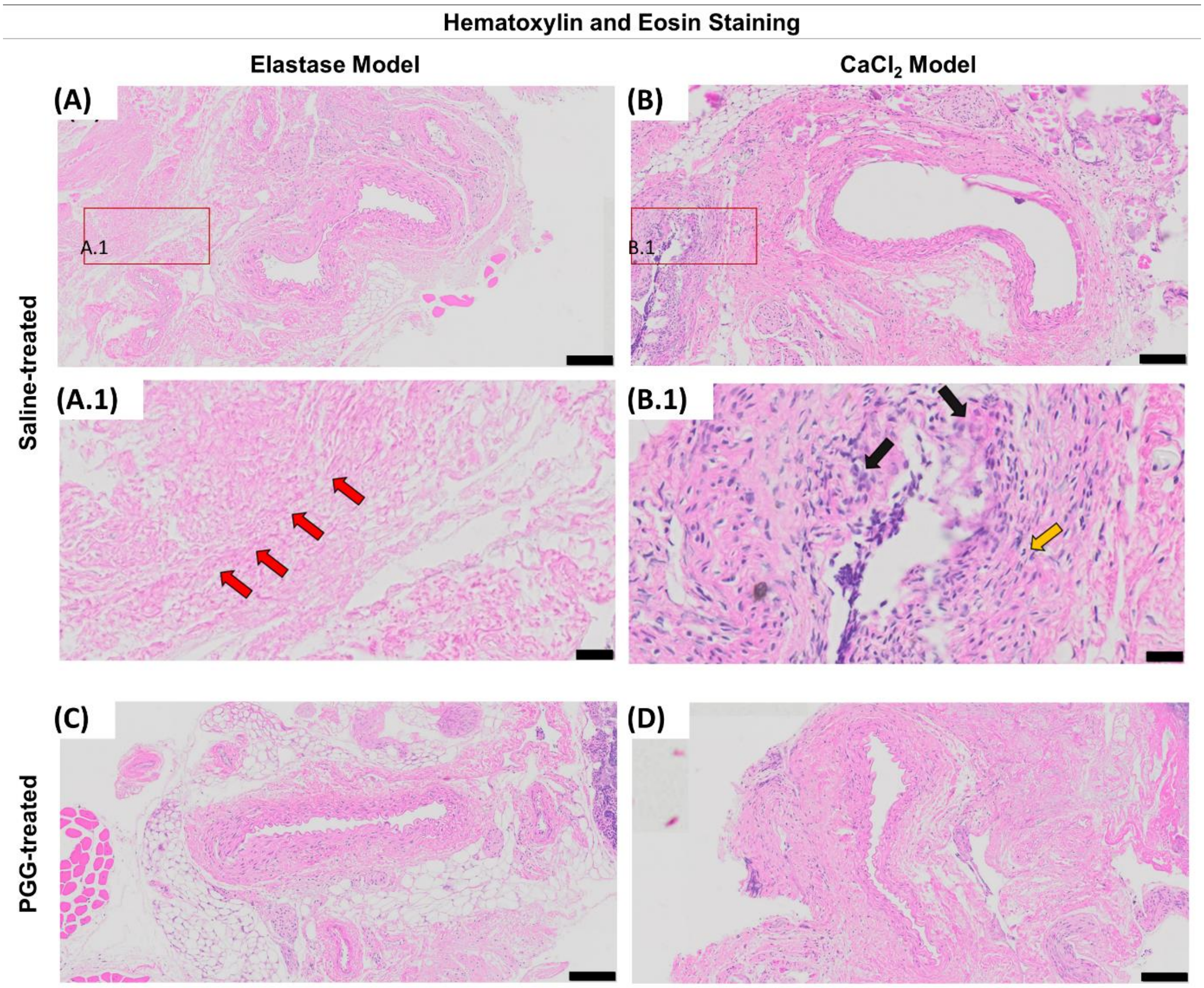

Histological characterization of explanted abdominal aortas. Hematoxylin and Eosin (H&E) staining of explanted abdominal aortas from the elastase (A and C) and $CaCl_2$ (B and D) injury models are shown here. Highly magnified regions of interest from saline-treated tissues from the elastase and $CaCl_2$ injury models (red box in images A and B) are shown in A.1 and B.1, respectively (magnification 40x; scale bar = 20µm). Tissue derived from the elastase model and further treated with saline, exhibited a heightened necrotic response with cells adjacent to the vessel demonstrating pyknosis and karyolysis outlined by a red box (A, magnification 10x; scale bar = 100µm) and identified in the higher magnification image by red arrows (A.1; magnification 40x; scale bar = 20µm). Corresponding cohort of animals in the elastase group treated with PGG did not exhibit a similar inflammatory characteristic (C). Further, the stained section of aortic tissues derived from $CaCl_2$ injury models treated with saline showed increased signs of chronic inflammation and macrophages infiltration adjacent to vessels (red box seen in B; magnification 10x; scale bar = 100µm), identified by black arrows in B.1 (magnification 40x; scale bar = 20µm). These tissues also exhibited some degree of fibrosis as well (yellow arrow seen in B.1). The extracellular microarchitecture was more preserved in their PGG-treated counterparts (D).

Table S1: Statistical analysis of biomechanical data obtained from pressure-inflation testing

The detailed results of the statistical analysis for the experimental groups (elastase + saline vs. Elastase + PGG, and $CaCl_2$ + Saline vs. $CaCl_2$ + PGG, respectively) are reported here.

Table S1. Statistical analysis of biomechanical data obtained from pressure-inflation testing

**Parameters**	**Groups**			
	Elastase + Saline	Elastase + PGG	$CaCl_2$ + Saline	$CaCl_2$ + PGG
Goodness of fit (R^2)	0.54	0.77	0.2	0.87
Slope of regression line	1.08	1.2	0.31	1.26
Are the slopes significantly different?	No (p=0.85)		No (p=0.37)	
Are the intercepts significantly different?	No (p=0.66)		No (p=0.72)	
