# Supplementary figures and images for: Animal Model Dependent Response to Pentagalloyl Glucose in Murine Abdominal Aortic Injury
Source: J Clin Med. 2021 Jan 9;10(2):219. doi: 10.3390/jcm10020219 (PMC7827576; doi:10.3390/jcm10020219)

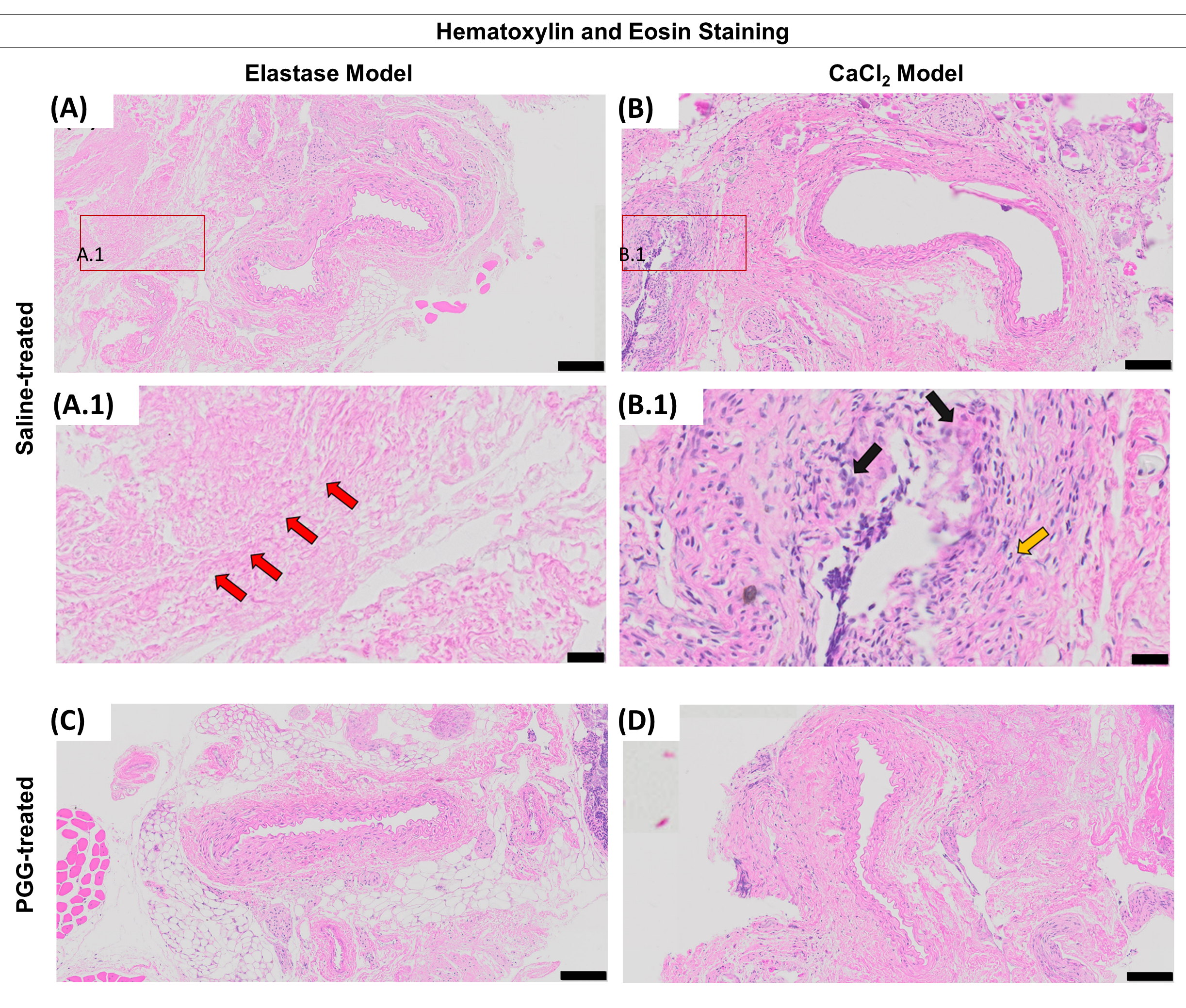

Supplement: Supplementary file 1 [file jcm-10-00219-s001.zip › Supplemental_FigureS1_Final.tif]
